# Burden of Ischemic Heart Disease in Central Asia from 1990 to 2021: A Systematic Analysis of the Global Burden of Disease Study 2021

**DOI:** 10.3390/ijerph23050675

**Published:** 2026-05-20

**Authors:** Dimash Davletov, Mukhtar Kulimbet, Alisher Makhmutov, Dinmukhammed Osser, Marat Pashimov, Batyrbek Assembekov, Kairat Davletov

**Affiliations:** 1 Atchabarov Scientific-Research Institute of Fundamental and Applied Medicine, Asfendiyarov Kazakh National Medical University, Almaty 050012, Kazakhstan; assembekov.b@kaznmu.kz (B.A.);; 2 Research Institute of Cardiology and Internal Diseases, Almaty 050000, Kazakhstan; mkbkul@gmail.com (M.K.);; 3 Ascension Saint Joseph Hospital, Chicago, IL 60657, USA; alshain9@gmail.com; 4 Danbury Hospital/Northwell, Danbury, CT 06810, USA; dinmukhammedosser@gmail.com; 5 Population Health Research Center, Al-Farabi Kazakh National University, Almaty 050040, Kazakhstan

**Keywords:** ischemic heart disease, Central Asia, mortality, incidence, prevalence, DALY

## Abstract

**Highlights:**

**Public health relevance—How does this work relate to a public health issue?**

- Ischemic heart disease (IHD) continues to be the leading cause of morbidity and mortality, placing a significant and rising burden on aging populations and healthcare systems around the world.
- The Central Asian region serves as a high-burden region with consistently and substantially elevated IHD incidences and mortality levels above global averages.

**Public health significance—Why is this work of significance to public health?**

- This paper highlights a key difference as while the global age-standardized incidences of IHD per 100,000 individuals have been declining from 419 to 373 between 1990 and 2021, age-standardized incidence rates per 100,000 people in the Central Asian region increased from 642 to 802 between 1990 and 2021.
- Whereas the age-standardized death rates per 100,000 and age-standardized DALY per 100,000 have decreased globally and also in Central Asia, there has been significant heterogeneity within the region.

**Public health implications—What are the key implications or messages for practitioners, policy makers and/or researchers in public health?**

- Addressing the IHD disparity necessitates a two-pronged strategy of effective primary prevention policies as well as increased interventions in interventional cardiology and secondary prevention.
- Given that countries in Central Asia have followed different paths based on health reforms, any intervention will need to be done based on country-specific epidemiology.

**Abstract:**

Ischemic heart disease (IHD) remains a leading cause of death globally. This study aims to analyze the burden of IHD in Central Asia and in individual countries of the region from 1990 to 2021, through a comparison of trends in incidence, prevalence, mortality, and disability-adjusted life years. Using data from the Global Burden of Disease (GBD) 2021 study, we extracted annual estimates for the Central Asian region, Kazakhstan, Kyrgyzstan, Uzbekistan, Tajikistan, Turkmenistan, Georgia, Armenia, Azerbaijan, and Mongolia. Metrics were reported as age-standardized rates per 100,000 population. Temporal trends were quantified using Average Annual Percent Change based on joinpoint regression models. A stratification by sex and age groups was done. Central Asia consistently maintained a higher IHD burden than the global average. While global age-standardized incidence rates per 100,000 population fell, Central Asia’s rates per 100,000 population rose from 641.97 in 1990 to 801.56 in 2021. Age-standardized death rates per 100,000 population in the region peaked in the mid-1990s following the dissolution of the Soviet Union but decreased overall from 320.47 in 1990 to 265.51 in 2021. However, this remains significantly higher than the 2021 global rate per 100,000 population of 108.73. Uzbekistan exhibited the highest growth in prevalence and incidence rates per 100,000 population, while Georgia demonstrated the largest reduction in DALYs rates per 100,000 population. Men demonstrated a higher burden across most metrics, although the sex gap narrowed in older populations. Central Asia faces rising incidence rates of IHD and burden levels that far exceed global averages. The significant heterogeneity among countries suggests that region-wide generalizations are insufficient and highlights the critical need for targeted, country-specific prevention programs and health system interventions.

## 1. Introduction

Ischemic heart disease (IHD) remains the world’s major cause of death and disability, with increasing absolute burden in aging and growing populations despite declining age-standardized rates in most high-income settings [1]. Worldwide evidence has documented substantial advances in IHD prevention and acute treatment in parts of Europe and East Asia during the past three decades but, despite these advances, improvement has been uneven, and some regions have fallen behind with high burden remaining [1,2].

Central Asia is one of those. National and Global Burden of Disease (GBD) studies regularly show that Central Asian countries have more than the world’s average IHD rates, and improvement has been more sluggish than in the majority of other regions since 1990. While a study in 2017 presented ischemic heart disease data from Central Asia, many global events, such as COVID-19, have occurred which may change the picture of epidemiology in the region [3]. 

This study aims to analyze the burden of ischemic heart disease in Central Asia from 1990 to 2021 using GBD data [4]. We will analyze the incidence, prevalence, mortality, DALYs, YLDs and YLLs trends for different ages and sexes in nine countries and Central Asia as a whole, comparing their trends to global estimates, to better understand the burden of ischemic heart disease in Central Asia.

## 2. Materials and Methods

### 2.1. Disease Definition

In the context of GBD 2021, ischemic heart disease (IHD) is defined as a condition characterized by reduced blood supply to the heart muscle, primarily resulting from atherosclerosis of the coronary arteries [5]. This category encompasses acute myocardial infarction, chronic stable angina, and chronic ischemic heart disease, adhering to the standard ICD diagnostic codes. The definition excludes non-ischemic cardiomyopathies and other cardiac conditions not primarily driven by coronary atherosclerosis, following the internal cause hierarchy established by GBD disease modeling protocols.

### 2.2. Data Acquisition and Metrics

We obtained annual estimates from 1990 to 2021 for incidence, prevalence, mortality, years of life lost (YLLs), years lived with disability (YLDs), and disability-adjusted life years (DALYs) associated with IHD. Data were extracted from the GBD 2021 database for individual countries such as Kazakhstan, Kyrgyzstan, Uzbekistan, Tajikistan, Turkmenistan, Georgia, Armenia, Azerbaijan, Mongolia, and Central Asia as a region. The GBD 2021 classifies these nine countries into the Central Asia region due to overall epidemiological homogeneity and geographical contiguity [6,7].

To ensure internal consistency across prevalence, incidence, and mortality estimates by year, location, sex, and age, the GBD utilizes the Bayesian-based Disease Modeling Meta-Regression (DisMod-MR) tool [5]. Mortality estimates specifically were generated using the Cause of Death Ensemble model (CODE) [8]. It is important to note that for the countries analyzed in this study, primary local data inputs regarding IHD were unavailable. Consequently, the GBD methodology utilized regional estimates and predictive covariates to model trends for these specific nations [5]. All metrics are reported as age-standardized rates per 100,000 population, standardized against the GBD reference population. Data were divided into a young adult group (ages 15–39) followed by standard 5-year age brackets ranging from 40–44 to 75–79 (40–44, 45–49, 50–54, 55–59, 60–64, 65–69, 70–74, 75–79) and elderly group including all ≥80 years.

To quantify temporal trends, we calculated the Average Annual Percent Change (AAPC). This metric represents the weighted average of annual percent changes derived from a joinpoint model. While this parsimonious approach provides a stable metric for long-term regional comparison, it is acknowledged that a zero-breakpoint model may smooth over significant short-term fluctuations, which are instead addressed through qualitative narrative analysis in the manuscript. Uncertainty was addressed by reporting 95% uncertainty intervals (UI), defined by the 2.5th and 97.5th percentiles of 1000 ordered draws.

### 2.3. Statistical Analysis

We conducted a descriptive analysis of age-standardized trends for incidence, prevalence, mortality, and DALYs, comparing Central Asian nations against global data. To detect significant trend shifts, we employed piecewise regression analysis. Based on the Bayesian Information Criterion (BIC) and the observation of relatively linear trajectories between 1990 and 2021, a model with zero breakpoints was selected. Consequently, AAPCs were estimated using log-linear models assuming a constant rate of change. Statistical significance was set at *p* < 0.05. All statistical computations and figures were executed using R software (version 4.3.0).

## 3. Results

### 3.1. Prevalence of Ischemic Heart Disease

**Figure 1:**
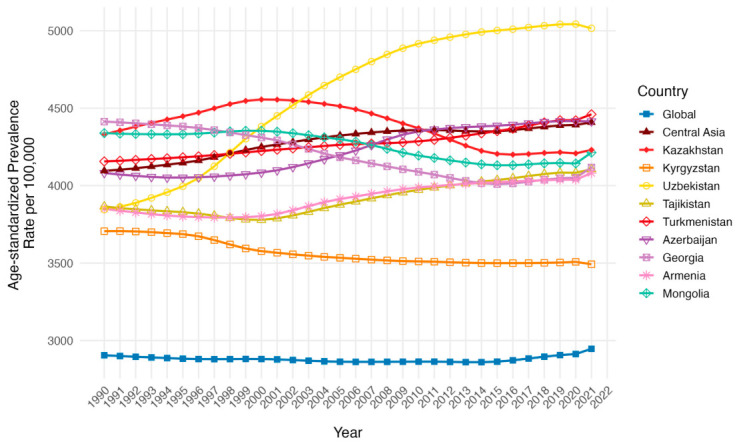
Temporal trends in Age-Standardized Prevalence Rates (ASPR) per 100,000 population across Central Asia as a region, Kazakhstan, Kyrgyzstan, Uzbekistan, Tajikistan, Turkmenistan, Georgia, Armenia, Azerbaijan, Mongolia and the global average 1990 to 2021 for both sexes.

Although the trends did not differ significantly, the age-standardized prevalence rate (ASPR) per 100,000 of the Central Asian region was higher than the global in 1990 (4095.9 [95% UI: 3802.3 to 4441.4] vs. 2904.7 [95% UI: 2576 to 3248.1]) and 2021 (4408.4 [95% UI: 4040.6 to 4801.1] vs. 2946.4 [95% UI: 2572.7 to 3424.3] in 2021) (Table 1, Figure 1). Notably, the highest positive slope in trend and growth in ASPR per 100,000 occurred in Uzbekistan from 3845.50 (95% UI: 3546.49 to 4202.85) in 1990 to 5015.59 (95% UI: 4660.91 to 5391.59) in 2021. While it was below the Central Asia region ASPR in 1990 initially, the 2021 ASPR per 100,000 was above the Central Asia region; a similar pattern was observed in Azerbaijan and Turkmenistan. In contrast to that, Kazakhstan, Georgia and Mongolia presented an opposite pattern with higher ASPR per 100,000 in 1990 and a decline below Central Asian value in 2021. Other countries with a growth of ASPR per 100,000 in 1990 and 2021 below the levels of Central Asia were Armenia and Tajikistan. Kyrgyzstan stands out as being the only country with a declining ASPR below the Central Asia ASPR per 100,000 in 1990 and 2021.

**Figure 2:**
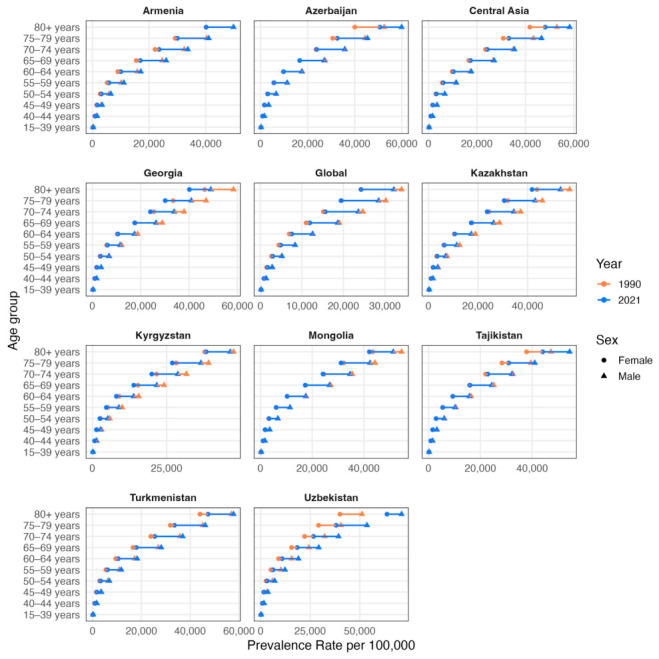
Comparison of crude prevalence rates per 100,000 population by sex and age groups (15–39, 40–44, 45–49, 50–54, 55–59, 60–64, 65–69, 70–74, 75–79, ≥80 years) in 1990 (orange) and 2021 (blue). Triangles denote male cohorts and circles denote female cohorts.

A male prevalence rate per 100,000 of IHD was presented across all Central Asian regions and globally in all age groups above 40 years (Figure 2). The population above 80 years had the highest prevalence in both sex groups. The change in trend due to growth between 1990 and 2021 started earliest in the age groups 50–55 in both sex groups in Uzbekistan, Armenia and Turkmenistan. Tajikistan and Azerbaijan showed a similar picture, starting with the age groups 75–79 and higher. On the other side, Georgia, Kyrgyzstan, Kazakhstan and Mongolia followed a trend of decreasing prevalence, consistent with the global pattern, especially in the male population.

### 3.2. Incidence of Ischemic Heart Disease

**Figure 3:**
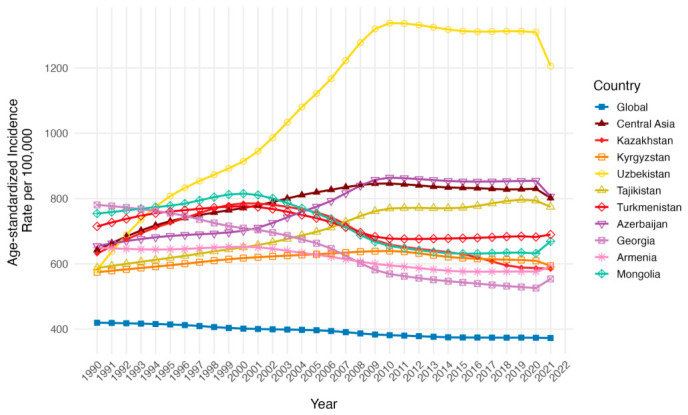
Temporal trends in Age-Standardized Incidence Rates (ASIR) per 100,000 population across Central Asia as a region, Kazakhstan, Kyrgyzstan, Uzbekistan, Tajikistan, Turkmenistan, Georgia, Armenia, Azerbaijan, Mongolia and the global average 1990 to 2021 for both sexes.

The age-standardized incidence rate (ASIR) per 100,000 in Central Asia rose from 641.97 (95% UI: 560.80 to 735.59) in 1990 to 801.56 (95% UI: 731.97 to 893.80) in 2021, while the global rates per 100,000 fell from 419.54 (95% UI: 351.07 to 498.15) in 1990 to 372.90 (95% UI: 307.95 to 444.19) in 2021, indicating a two-fold difference in 2021 (Table 1, Figure 3). Countries above the Central Asia ASIR per 100,000 in 1990, like Armenia, Georgia, Mongolia, Turkmenistan, presented a decline in 2021 with some reaching plateau. Primarily being below Central Asian ASIR per 100,000, Kazakhstan had a rising trend towards the 2000s with a steady drop up to 2021 to levels below the initial presentation. On the other hand, countries with similar initial presentations in 1991, Kyrgyzstan and Tajikistan, showed growth towards 2021, still below the Central Asian ASIR per 100,000. Azerbaijan had a pattern similar to the Central Asian region. Strikingly, Uzbekistan, from having the least ASIR per 100,000 in 1990 turned out as the country with the largest ASIR per 100,000 in 2021, with the highest drop in the same year after a ten-year plateau.

**Figure 4:**
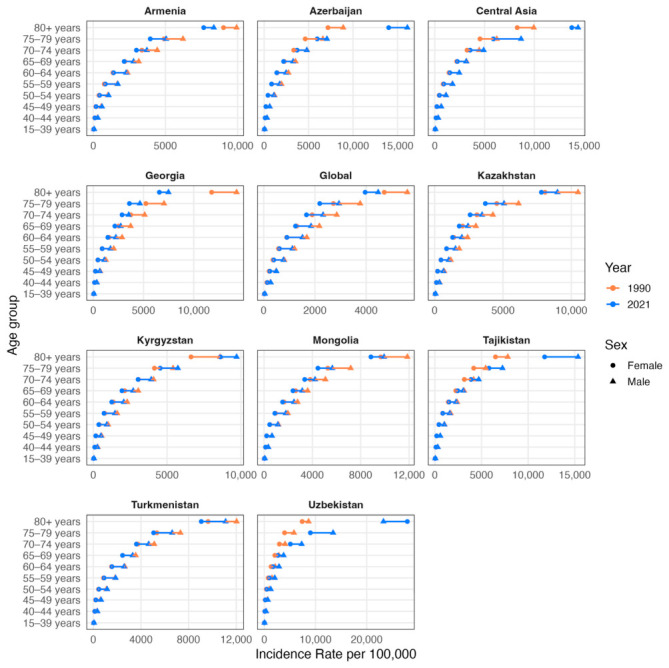
Comparison of crude incidence rates per 100,000 population by sex and age groups (15–39, 40–44, 45–49, 50–54, 55–59, 60–64, 65–69, 70–74, 75–79, ≥80 years) in 1990 (orange) and 2021 (blue). Triangles denote male cohorts and circles denote female cohorts.

The incidence rate per 100,000 was equally similar in most age groups of both sexes with some exceptions of male incidence occurring more (Figure 4). An increase in incidence rate per 100,000 from 1990 to 2021 was observed clearly in age groups over 70 in Uzbekistan, Tajikistan, Azerbaijan and Kyrgyzstan. Incidence rate per 100,000 in other countries decreased in the same period, mostly starting from 60 years old with the highest differences in Georgia.

### 3.3. Death Rates of Ischemic Heart Disease

**Figure 5:**
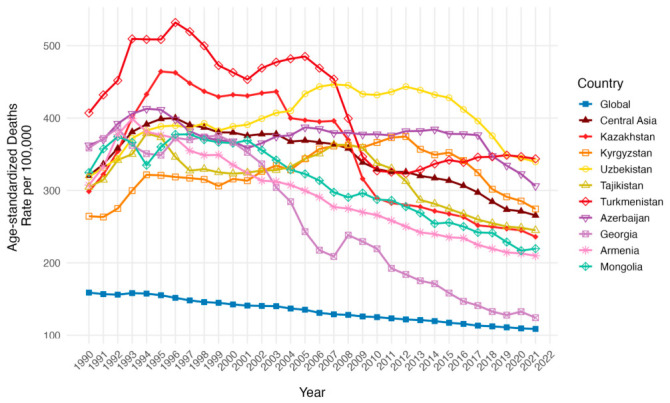
Temporal trends in Age-Standardized Death Rates (ASDR) per 100,000 population across Central Asia as a region, Kazakhstan, Kyrgyzstan, Uzbekistan, Tajikistan, Turkmenistan, Georgia, Armenia, Azerbaijan, Mongolia, and the global average from 1990 to 2021 for both sexes.

The Central Asia region had a decrease in age-standardized death rates (ASDR) per 100,000 from 320.47 (95% UI: 299.83 to 332.36) in 1990 to 265.51 (95% UI: 240.67 to 290.42) in 2021, even though the trend had a rise in the first half of 1990 (Table 2, Figure 5). The global trend was lower in comparison and had a steady negative slope from 158.90 (95% UI: 148.14 to 165.30) in 1990 to 108.73 (95% UI: 99.60 to 115.38) in 2021 with a significant AAPC change of −1.93 (95% UI: −3.75 to −0.08). All countries pictured a growth trend from 1990 with a peak around 1995. Uzbekistan, Kyrgyzstan, Azerbaijan and Tajikistan held the momentum of growth up to the 2010s and only Azerbaijan and Tajikistan returned to levels below 1990 in the ASDR per 100,000 of 2021. Turkmenistan and Kazakhstan, the leaders in the peak of 1995, showed slow decline with a sharp fall in 2008 with a division in 2010 where Kazakhstan continued the decline. Mongolia, Armenia and Georgia demonstrated a negative slope starting from 1995 with a fluctuation in Georgia ASDR per 100,000 in 2008.

**Figure 6:**
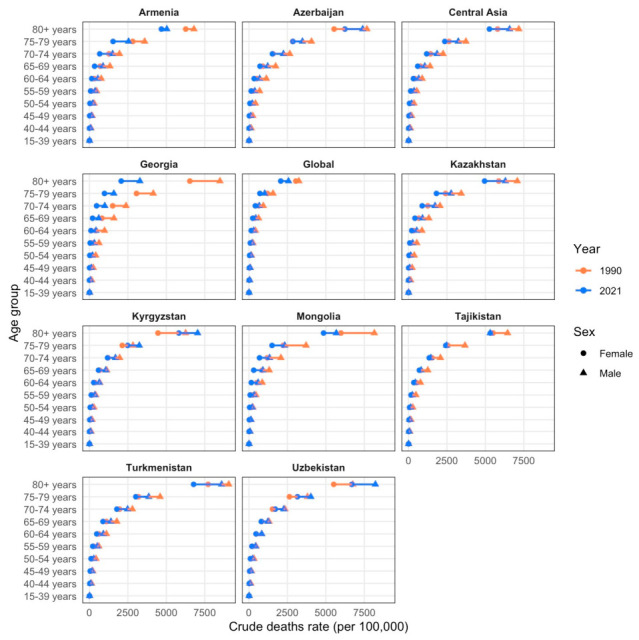
Comparison of crude death rates per 100,000 population by sex and age groups (15–39, 40–44, 45–49, 50–54, 55–59, 60–64, 65–69, 70–74, 75–79, ≥80 years) in 1990 (orange) and 2021 (blue). Triangles denote male cohorts and circles denote female cohorts.

Ischemic heart disease mortality rates per 100,000 population lowered in all age groups for both sexes in all countries from 1990 to 2021 (Figure 6). An exception for both sexes was in the ages over 75 in Uzbekistan and Kyrgyzstan. Females in the age groups 70–74 in Uzbekistan and 80+ in Azerbaijan also had a similar tendency. Tajikistan had no difference in sex mortality in 2021.

### 3.4. DALY Rates of Ischemic Heart Disease

**Figure 7:**
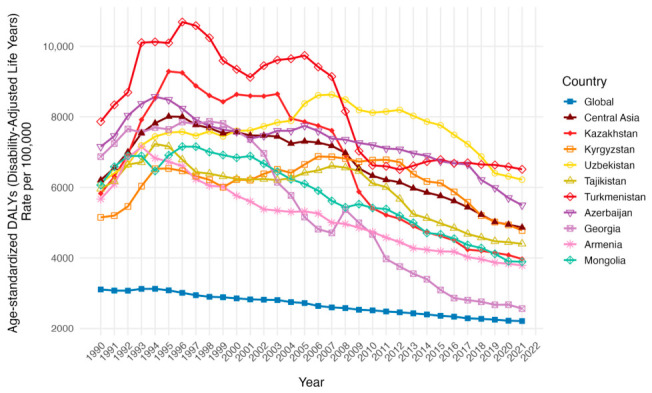
Temporal trends in age-standardized DALY Rates per 100,000 population across Central Asia as a region, Kazakhstan, Kyrgyzstan, Uzbekistan, Tajikistan, Turkmenistan, Georgia, Armenia, Azerbaijan, Mongolia and the global average from 1990 to 2021 for both sexes.

The age-standardized disability-adjusted life years (DALY) rates per 100,000 population trends was higher in Central Asia in comparison with global and both had a trend of decreasing from 6206.85 (95% UI: 5937.37 to 6407.43) and 3107.61 (95% UI: 2966.50 to 3222.67) in 1990 to 4864.49 (95% UI: 4415.55 to 5338.75) and 2212.16 (95% UI: 2075.54 to 2327.61) in 2021 respectively (Table 2, Figure 7). Moreover, the AAPC in the global trend was decreasing steadily (−1.94, 95% UI: −3.45 to −0.20). All countries besides Uzbekistan had a decrease in age-standardized DALY rates per 100,000 population from 1990 to 2021, Georgia having the largest decrease from 6871.09 (95% UI: 6545.28 to 7157.65)—being above Central Asia in 1990—to 2565.24 (95% UI: 2301.90 to 2822.20) in 2019, almost reaching global age-standardized DALY rates per 100,000 population. Among countries with the highest AAPC were Armenia (−2.66, 95% UI: −4.76 to −0.53) and Kyrgyzstan (−2.02, 95% UI: −3.87 to −0.13), both being above the global levels (−1.84, 95% UI: −3.45 to −0.20). Uzbekistan demonstrated a little growth in age-standardized DALY rates per 100,000 population from 6122.18 (95% UI: 5815.24 to 6372.06) in 1990 to 6218.80 (95% UI: 5449.76 to 7058.07) in 2021, with a negative AAPC (−0.75, 95% UI: −3.30 to 1.88).

**Figure 8:**
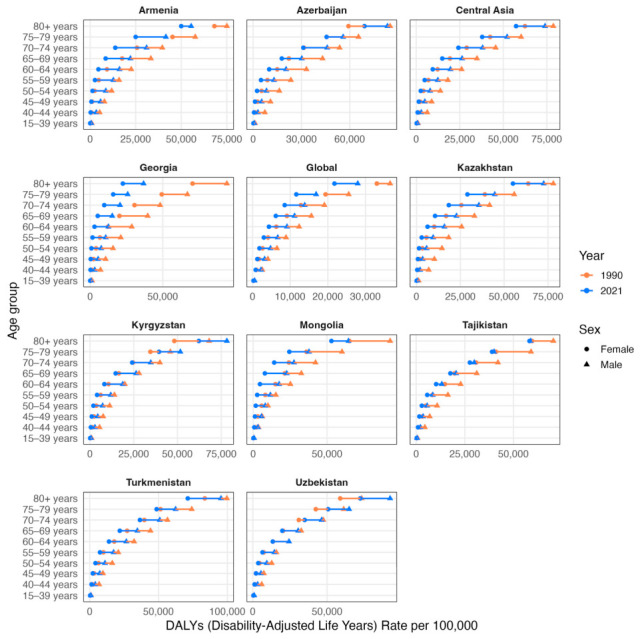
Comparison of crude DALY rates per 100,000 population by sex and age groups (15–39, 40–44, 45–49, 50–54, 55–59, 60–64, 65–69, 70–74, 75–79, ≥80 years) in 1990 (orange) and 2021 (blue). Triangles denote male cohorts and circles denote female cohorts.

DALY rates per 100,000 population were higher predominantly in males in all years, countries and age-groups (Figure 8). Females in the age groups over 80 years in Azerbaijan and 70–74 in Uzbekistan had higher DALY rates per 100,000 population in 2020 compared to 1990. A similar pattern was observed for both sexes in age groups over 75–79 and 80+ in Kyrgyzstan and Uzbekistan. Other countries lowered DALY rates per 100,000 population in other age groups. Notably, DALY rates per 100,000 population in 2021 for Tajikistan was similar for both sexes.

### 3.5. YLL and YLD of Ischemic Heart Disease

**Figure 9:**
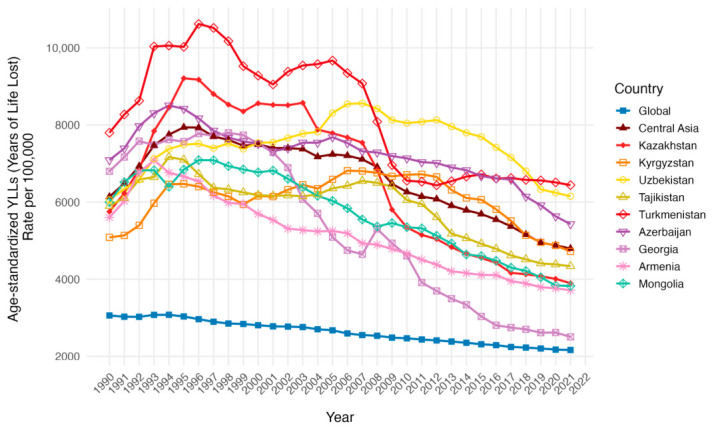
Temporal trends in age-standardized YLL Rates per 100,000 population across Central Asia as a region, Kazakhstan, Kyrgyzstan, Uzbekistan, Tajikistan, Turkmenistan, Georgia, Armenia, Azerbaijan, Mongolia and the global average from 1990 to 2021 for both sexes.

**Figure 10:**
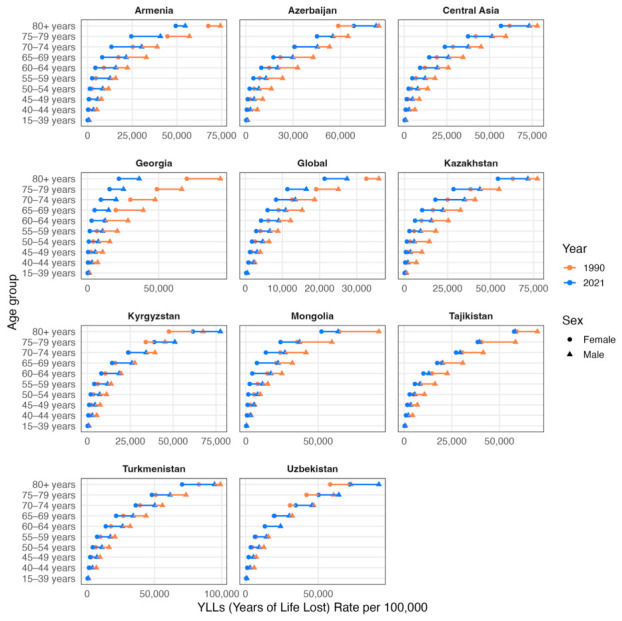
Comparison of crude YLL rates per 100,000 population by sex and age groups (15–39, 40–44, 45–49, 50–54, 55–59, 60–64, 65–69, 70–74, 75–79, ≥80 years) in 1990 (orange) and 2021 (blue). Triangles denote male cohorts and circles denote female cohorts.

**Figure 11:**
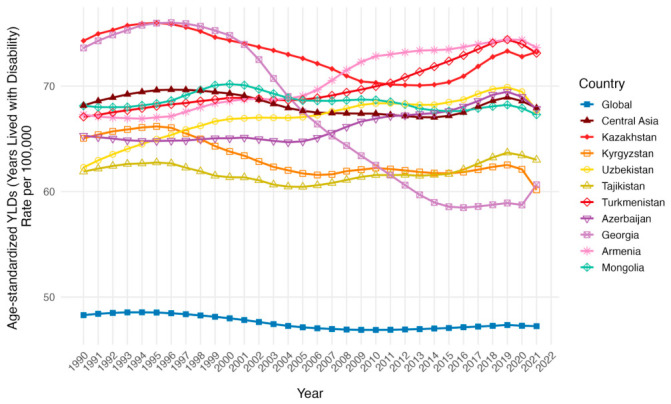
Temporal trends in age-standardized YLD Rates per 100,000 population across Central Asia as a region, Kazakhstan, Kyrgyzstan, Uzbekistan, Tajikistan, Turkmenistan, Georgia, Armenia, Azerbaijan, Mongolia and the global average from 1990 to 2021 for both sexes.

The age-standardized years of life lost (YLL) rates per 100,000 population trend and numbers did not differ significantly from those of DALY in all countries (Table 3, Figure 9 and Figure 10). However, the age-standardized years lived with disability (YLD) rates per 100,000 population presented a drop in global rates from 48.28 (95% UI: 31.82 to 66.87) in 1990 to 47.24 (95% UI: 31.00 to 65.31) in 2021, and larger rates in Central Asia from 68.16 (95% UI: 44.48 to 94.15) in 1990 to 67.92 (95% UI: 44.20 to 94.10) in 2021 (Figure 11). Armenia, Azerbaijan, Tajikistan, Turkmenistan and Uzbekistan’s age-standardized YLD rates rose from 1990 to 2021, but only Turkmenistan demonstrated a positive AAPC (0.48, 95% UI: −1.23 to 2.22). While the other countries followed a lowering of age-standardized YLD rates per 100,000 population from 1990 to 2021, only Kazakhstan was above the Central Asia values in both 1990 and 2021 (74.29 [95% UI: 48.52 to 104.10] and 73.22 [95% UI: 47.04 to 102.74]). 

**Figure 12:**
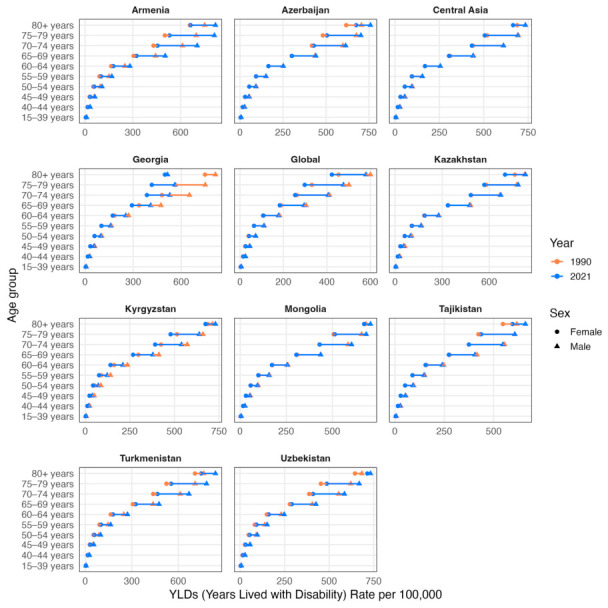
Comparison of crude YLD rates per 100,000 population by sex and age groups (15–39, 40–44, 45–49, 50–54, 55–59, 60–64, 65–69, 70–74, 75–79, ≥80 years) in 1990 (orange) and 2021 (blue). Triangles denote male cohorts and circles denote female cohorts.

A tendency for alignment of sexes is observed in age groups above 80 years after a steady growth of distance between sexes starting from age groups 40–44 (Figure 12). The main differences in YLD rates per 100,000 population starts from age 70 and above, where all Central Asian countries had 2021 rates around 400 for women and 600 for men while the global YLD rates per 100,000 population were around 200 for women and 400 for men. Of all countries, Georgia presented the highest lowering of YLD rates in these age groups from 1990 to 2021. Kazakhstan, Azerbaijan and Turkmenistan had the highest YLD rates per 100,000 population in these age groups among both sexes.

## 4. Discussion

Across three decades, Central Asia carried a consistently higher IHD burden than the global average. The ASPR in Central Asia remained elevated in both 1990 (4095.9 per 100,000) and 2021 (4408.4 per 100,000), substantially exceeding the corresponding global figures (2904.7 and 2946.4 per 100,000, respectively). Incidence moved in opposite directions—ASIR declined globally from 419.5 to 372.9 per 100,000 but rose in Central Asia from 642.0 to 801.6 per 100,000—whereas age-standardized death rates fell in both settings (globally from 158.9 to 108.7; Central Asia from 320.5 to 265.5 per 100,000) yet remained approximately 2.4-fold higher in Central Asia in 2021. Among individual countries, Uzbekistan diverged most markedly, with ASPR rising from 3845.5 to 5015.6 and ASIR more than doubling from 582.0 to 1206.0 per 100,000. Kyrgyzstan was the only country with a declining ASPR (AAPC −1.23), and Georgia achieved the largest DALY reduction (from 6871.1 to 2565.2 per 100,000). Men bore a higher burden across outcomes and ages, with the gap narrowing only at the oldest ages.

The temporal patterns observed—most notably, the peak in IHD mortality in 1994–1996—are likely closely linked to the socio-political instability that followed the dissolution of the Soviet Union. The Central Asian republics started the 1990s in economic collapse; GDP plummeted, healthcare investment disappeared, and millions were pushed into unemployment and poverty. Brainerd et al. attributed Central Asia’s rising IHD burden to the 1990s economic crisis, which would have manifested in several likely ways [9,10]. First, health systems were severely destabilized; experienced clinicians emigrated, medical supply chains were broken, and prevention programs stopped [11]. The acute cardiac care capacity of hospitals deteriorated at a time when demand and stress intensified. Second, the population’s risk factor profile deteriorated in the short term. The acute socio-economic strain, uncertainty, and psychosocial stress may precipitate cardiac events. More specifically, the elimination of Soviet state controls led to increased access to tobacco and alcohol [12]. Per capita consumption of alcohol in Russia and likely in Central Asia surged in the early 1990s after the elimination of price controls, which fueled the increases in cardiovascular and also external-cause mortality. Diets also potentially worsened in the short term—with poverty, individuals resorted to cheaper high-starch and salty preserved foods and were unable to afford fruit or lean protein [13]. All these factors might have contributed to the mid-90s mortality peak that we see in the data.

Uzbekistan stands out for a steady rise in IHD burden. Its ASIR rose from 582.0 to 1206.0 per 100,000 and its ASDR increased from 318.6 to 339.5 per 100,000 between 1990 and 2021, making Uzbekistan the only Central Asian country where the death rate rose over the study period. A meta-analysis reported very high population-level salt consumption in this country, which is consistent with the observed rising incidence and prevalence trends [14].

Georgia implemented comprehensive smoke-free and tobacco-control laws in 2017 with evidenced reductions in acute myocardial infarction incidence and indoor SHS exposure, and achieved progress toward Universal Health Coverage in 2013—both in line with enhanced CVD prevention/acute care access and decreasing burden [15,16]. Indeed, Georgia’s age-standardized DALY rate dropped from 6871.1 to 2565.2 per 100,000 (AAPC −1.16), approaching the 2021 global average of 2212.2 per 100,000 and representing the largest absolute DALY reduction in the region. The variability in 2008 is plausibly explained by the short, high-intensity Russian–Georgian war and related service disruptions [17]. Additionally, Kazakhstan scaled interventional cardiology rapidly in the 2010–2015 period, shortening door-to-device times and expanding PCI networks—changes that typically reduce fatality and post-MI disability and, over time, prevalence [18]. Correspondingly, Kazakhstan’s ASDR declined from 298.7 to 236.0 per 100,000 (AAPC −0.86) and its ASPR fell from 4331.5 to 4230.9 per 100,000. Very high salt intake remains a concern, but health system investments appear to have contributed to mortality reductions [19].

Armenia also merits attention; it recorded the only statistically significant decline in ASDR among individual Central Asian countries (AAPC −2.76, 95% CI: −5.11 to −0.35), with its death rate falling from 307.4 to 209.8 per 100,000 and DALY rate from 5669.8 to 3788.5 per 100,000 (AAPC −2.66). Azerbaijan and Turkmenistan, by contrast, maintained some of the highest absolute burden levels in the region: Turkmenistan’s ASDR remained at 343.7 per 100,000 in 2021 with a near-zero AAPC (0.15), and Azerbaijan’s ASDR of 306.1 per 100,000 showed only modest decline (AAPC −0.58). These contrasts underscore that, even within Central Asia, countries at similar stages of economic development have diverged considerably in their IHD trajectories, likely reflecting differences in the pace of health system reform, tobacco and alcohol policy, and dietary transitions.

Our analysis confirms well-known but important patterns in IHD epidemiology: rates increase exponentially with age and have historically been higher in men than women [20]. In Central Asia, IHD incidence and prevalence remained low in young adults, then rose sharply from middle-age onward, peaking in the oldest cohorts. This mirrors global observations that atherosclerotic cardiovascular disease is relatively rare before age 40, due to the time required for risk factors to exert their arterial damage. After age 40–50, cumulative exposures reach a threshold where clinical IHD manifests, hence the steep climb in both men and women. By age 70+, a large proportion of both sexes have some degree of coronary artery disease, which explains why the sex gap diminishes in older age groups—women essentially “catch up” after menopause as the protective effects of estrogen wane and if they have similar lifespans their risk begins to approach that of men [21,22]. Additionally, men who are extremely susceptible may die in middle age, leaving a cohort of relatively hardier male survivors at older ages, which also makes the surviving men’s average risk more similar to women’s.

In Central Asia, these general trends are present, but certain nuances emerge. The male excess in IHD burden is pronounced in early adult and middle ages. For example, we observed much higher ASIR and ASDR for men in the 40–59 range. This is largely attributable to behavioral risk differences: men have historically used tobacco and alcohol far more than women in this region, and men may experience more work-related stress and have higher rates of untreated hypertension [23,24,25]. Cultural factors also mean women might underreport symptoms or have less exposure to some risks, delaying their IHD onset [25]. Consequently, men suffer myocardial infarctions and sudden cardiac deaths at younger ages, which inflate the male ASDR in those age bands. By older ages, however, many of those high-risk men have already been taken by cardiovascular or other causes, and women who have lived that long often have significant risk accumulations. This results in the narrowing sex mortality gap we and others have noted in late life.

Tajikistan’s virtually nonexistent absence of male surplus in 2021 is an exceptional case. Smoking and alcohol use in Tajikistan, being lower in comparison with other countries, may attenuate two of the strongest IHD risk factors that typically place men at greater jeopardy [23,26]. In addition, Tajikistan’s women are likely to have greater indoor air pollution due to burning solid fuels, leading to chronic inflammation and hypertension in women using firewood as fuel—a specific issue in rural Tajik and Afghan populations [27,28]. It is also possible that male labor migration artificially lowers the observed male IHD rates in the local data [28].

A notable observation in our data is the sudden fluctuations in incidence and prevalence around 2021, particularly in Uzbekistan (where ASIR dropped sharply after a decade-long plateau) and in several countries where prevalence trends reversed direction. One potential explanation, excluding true intervention effect, is that the COVID-19 pandemic altered cause-of-death patterns and health-seeking in that year [29]. In most nations, individuals postponed medicine appointments and elective procedures for the heart in 2020–2021, something that may temporarily lower IHD reported incidence [30]. At the same time, some deaths that may have otherwise been attributed to IHD in prior years were in 2020–21 explained by COVID-19 if infected, possibly creating an artifact of decreased IHD statistics [31].

### 4.1. Policy Implications

These findings carry several policy implications. Countries with persistently rising IHD incidence, such as Uzbekistan, Tajikistan, and Azerbaijan, require urgent investment in primary prevention—particularly population-wide salt reduction strategies, tobacco control legislation, and opportunistic screening for hypertension and dyslipidemia within primary care. Georgia’s experience demonstrates that comprehensive tobacco-control laws combined with universal health coverage can produce measurable reductions in IHD burden within a relatively short timeframe, providing a potential model for neighboring countries. Kazakhstan’s expansion of interventional cardiology networks illustrates the complementary role of acute care investment in reducing case fatality. A region-wide approach that combines these strategies—strengthening both upstream prevention and downstream acute and secondary care—while tailoring priorities to each country’s specific burden profile, offers the most promising path toward reducing the persistent IHD gap between Central Asia and the rest of the world [32].

### 4.2. Strengths

To our knowledge, this is the only comprehensive epidemiological research of ischemic heart disease in Central Asia from 1990 to 2021 with the usage of data from the GDB 2021 study. Examining prevalence, incidence, mortality, DALYs, YLLs, YLDs trends and AAPC over three decades and by age and sex provided a broad, disaggregated view of both fatal and non-fatal IHD burden and highlighted vulnerable subgroups for targeted action.

### 4.3. Limitations

Data from Central Asia is heterogeneous: vital-registration and surveillance systems collapsed or were incomplete in the 1990s and remain variable, so many estimates rely on sparse primary data, modeling and extrapolation and are subject to misclassification of causes of death. Therefore, our study was subject to all the limitations discussed in previous GBD publications [33,34,35,36]. Some data can be unreliable due to the tendency of statistics being more positive for achieving governmental tasks in some countries [37]. DALYs depend on standardized disability weights and assumptions that may not fully capture local experience due to sparse data. Sparse data can produce artefactual peaks or troughs and wide uncertainty intervals that were shown often in our study. While we identified broad trends in IHD burden, this study did not include a formal analysis of individual risk factor attribution, such as the specific contributions of hypertension, diabetes, or obesity. Although the GBD framework identifies high systolic blood pressure, dietary sodium, tobacco use, and high LDL cholesterol as the leading modifiable risk factors for IHD globally, future studies incorporating specific regional risk factor data are needed to prioritize the most impactful interventions [38,39]. Recent global analyses have demonstrated that resource-limited regions face a growing IHD burden driven particularly by rising fasting plasma glucose and body mass index [40], a pattern that may be increasingly relevant to the rapidly urbanizing Central Asian populations. The current analysis utilizes the broad GBD category for ischemic heart disease, which does not distinguish between acute coronary events and chronic manifestations. This lack of clinical granularity may mask differences in how health systems manage acute care versus long-term secondary prevention. The extension of this analysis to 2021 captures the confounding influence of the COVID-19 pandemic. The pandemic altered healthcare-seeking behavior—major cardiac centers reported a 37% decrease in total cardiac surgeries and a 23–26% fall in cardiovascular hospitalizations during the peak pandemic months [41].

Although the use of a zero-breakpoint joinpoint model to calculate AAPCs provides a clear overview of the 31-year burden trajectory, there is an inherent tension between this statistical simplification and the non-linear historical realities of the region. The distinct mortality peaks of the mid-1990s, and the observed acute fluctuations, represent significant deviations from a constant rate of change. While a model permitting breakpoints might capture these specific events with greater statistical sensitivity, we prioritized the zero-breakpoint AAPC to maintain a uniform basis for comparison between countries with heterogeneous data quality. By complementing the statistical trend with a detailed narrative of these socio-political and public health disruptions, we aim to provide a more holistic representation of the IHD burden than a statistical model alone could convey.

## 5. Conclusions

This comprehensive analysis of GBD data from 1990 to 2021 reveals that Central Asia carries an IHD burden that consistently and significantly exceeds the global average. A critical divergence is observed in age-standardized incidence rates—while global rates per 100,000 population have declined, rates in Central Asia continue to rise. Although mortality and DALYs have generally decreased, these metrics remain far above global standards as of 2021. Consequently, these findings highlight the persistent challenge of IHD in Central Asia and the critical need for targeted, country-specific health interventions. Policy efforts should prioritize population-wide salt reduction strategies, particularly in high-consumption settings like Uzbekistan, alongside the implementation of rigorous tobacco control legislation as modeled by Georgia. Furthermore, strengthening primary care infrastructure to enhance opportunistic screening and management of hypertension and dyslipidemia is essential to bridge the gap in secondary prevention. Finally, addressing the persistent IHD challenge in Central Asia requires a dual approach that integrates robust upstream preventive policies with the continued expansion of access to acute and interventional cardiology services.

## Figures and Tables

**Table 1:** Age-standardized prevalence and age-standardized incidence rates (per 100,000) of ischemic heart disease for both sexes from 1990 to 2021 and Average Annual Percent Changes (AAPCs).

	Age-Standardized Prevalence Rates (95% UI)			Age-Standardized Incidence Rates (95% UI)		
	1990	2021	AAPC 1990–2021 (95% CI)	1990	2021	AAPC 1990–2021 (95% CI)
Global	2904.72 (2575.99 to 3248.10)	2946.38 (2572.69 to 3424.32)	−0.43 (−2.44 to 1.61)	419.54 (351.07 to 498.15)	372.90 (307.95 to 444.19)	−1.03 (−3.10 to 1.08)
Central Asia	4095.93 (3802.28 to 4441.37)	4408.38 (4040.59 to 4801.11)	−0.42 (−2.73 to 1.94)	641.97 (560.80 to 735.59)	801.56 (731.97 to 893.80)	−0.10 (−2.56 to 2.43)
Armenia	3850.37 (3557.26 to 4182.44)	4081.36 (3674.11 to 4505.40)	−0.62 (−3.06 to 1.88)	649.55 (547.43 to 772.11)	591.00 (490.42 to 710.55)	−1.73 (−4.20 to 0.81)
Azerbaijan	4081.71 (3790.91 to 4400.17)	4415.23 (4047.75 to 4805.90)	−0.26 (−2.60 to 2.13)	654.64 (567.56 to 759.47)	806.26 (725.60 to 906.90)	0.38 (−2.27 to 3.10)
Georgia	4413.48 (4088.32 to 4764.84)	4117.67 (3704.65 to 4551.63)	−0.40 (−2.39 to 1.62)	780.37 (695.73 to 888.33)	553.85 (458.86 to 662.12)	−0.51 (−2.58 to 1.60)
Kazakhstan	4331.53 (4033.68 to 4698.23)	4230.88 (3828.87 to 4676.98)	−0.68 (−3.04 to 1.75)	631.54 (553.86 to 718.39)	584.39 (515.35 to 677.98)	−0.61 (−2.99 to 1.84)
Kyrgyzstan	3706.04 (3415.29 to 4033.23)	3492.31 (3183.66 to 3858.39)	−1.23 (−3.36 to 0.95)	574.33 (468.95 to 692.84)	594.10 (510.66 to 697.78)	−1.33 (−3.58 to 0.97)
Mongolia	4339.07 (3986.59 to 4702.99)	4213.77 (3806.63 to 4658.65)	−0.17 (−2.28 to 1.99)	754.07 (614.27 to 912.76)	669.06 (547.48 to 799.77)	−0.09 (−2.36 to 2.23)
Tajikistan	3865.59 (3560.11 to 4190.30)	4105.81 (3730.25 to 4525.27)	−0.38 (−2.60 to 1.90)	587.55 (486.50 to 715.97)	774.65 (678.93 to 883.79)	0.60 (−1.71 to 2.97)
Turkmenistan	4156.32 (3830.23 to 4481.95)	4460.74 (4029.37 to 4943.29)	0.45 (−1.82 to 2.78)	714.65 (598.11 to 837.12)	689.66 (588.79 to 796.61)	0.29 (−2.05 to 2.69)
Uzbekistan	3845.50 (3546.49 to 4202.85)	5015.59 (4660.91 to 5391.59)	0.01 (−2.45 to 2.53)	581.99 (511.67 to 667.68)	1206.01 (1125.02 to 1305.45)	0.67 (−2.16 to 3.58)

**Table 2:** Age-standardized death and age-standardized DALY rates (per 100,000) of ischemic heart disease for both sexes from 1990 to 2021 and Average Annual Percent Change (AAPC).

	Age-Standardized Death Rates per 100,000 Population (95% UI)			Age-Standardized Disability-Adjusted Life Years (DALY) Rates per 100,000 Population (95% UI)		
	1990	2021	AAPC 1990–2021 (95% CI)	1990	2021	AAPC 1990–2021 (95% CI)
Global	158.90 (148.14 to 165.30)	108.73 (99.60 to 115.38)	−1.93 (−3.75 to −0.08)	3107.61 (2966.50 to 3222.67)	2212.16 (2075.54 to 2327.61)	−1.84 (−3.45 to −0.20)
Central Asia	320.47 (299.83 to 332.36)	265.51 (240.67 to 290.42)	−0.87 (−3.23 to 1.55)	6206.85 (5937.37 to 6407.43)	4864.49 (4415.55 to 5338.75)	−1.18 (−3.30 to 0.98)
Armenia	307.44 (286.83 to 323.83)	209.78 (186.39 to 233.39)	−2.76 (−5.11 to −0.35)	5669.83 (5377.99 to 5942.52)	3788.46 (3403.75 to 4233.65)	−2.66 (−4.76 to −0.53)
Azerbaijan	362.31 (337.11 to 388.22)	306.13 (270.78 to 343.48)	−0.58 (−3.66 to 2.61)	7154.02 (6698.23 to 7635.33)	5496.28 (4818.68 to 6182.32)	−1.08 (−3.80 to 1.72)
Georgia	358.65 (337.55 to 374.96)	124.16 (111.04 to 136.51)	−0.80 (−2.80 to 1.24)	6871.09 (6545.28 to 7157.65)	2565.24 (2301.90 to 2822.20)	−1.16 (−2.95 to 0.66)
Kazakhstan	298.65 (276.16 to 319.98)	235.98 (212.04 to 258.87)	−0.86 (−3.15 to 1.49)	5827.04 (5426.21 to 6249.33)	3970.50 (3569.75 to 4401.12)	−1.38 (−3.40 to 0.69)
Kyrgyzstan	264.38 (242.39 to 284.65)	274.41 (234.16 to 313.72)	−1.94 (−4.08 to 0.24)	5149.84 (4726.51 to 5557.72)	4780.48 (4093.58 to 5490.56)	−2.02 (−3.87 to −0.13)
Mongolia	324.79 (286.63 to 365.71)	219.59 (190.33 to 247.40)	−1.22 (−3.24 to 0.85)	6067.42 (5273.21 to 6935.77)	3892.31 (3366.09 to 4387.10)	−1.22 (−3.12 to 0.71)
Tajikistan	304.83 (266.01 to 336.17)	244.92 (206.56 to 281.62)	−1.06 (−3.39 to 1.33)	5959.98 (5301.73 to 6541.09)	4400.38 (3740.14 to 5106.84)	−1.31 (−3.40 to 0.82)
Turkmenistan	407.01 (383.66 to 423.46)	343.68 (280.94 to 420.00)	0.15 (−2.12 to 2.47)	7863.83 (7511.22 to 8203.80)	6512.70 (5219.52 to 8027.65)	0.15 (−1.85 to 2.20)
Uzbekistan	318.62 (295.07 to 333.63)	339.48 (295.64 to 382.01)	−0.40 (−3.28 to 2.57)	6122.18 (5815.24 to 6372.06)	6218.80 (5449.76 to 7058.07)	−0.75 (−3.30 to 1.88)

**Table 3:** Age-standardized YLL and age-standardized YLD rates per 100,000 population of ischemic heart disease for both sexes from 1990 to 2021 and Average Annual Percent Change (AAPC).

	Years of Life Lost (YLL) Rates per 100,000 Population (95% UI)			Years Lived with Disability (YLD) Rates per 100,000 Population (95% UI)		
	1990	2021	AAPC 1990–2021 (95% CI)	1990	2021	AAPC 1990–2021 (95% CI)
Global	3059.32 (2924.75 to 3170.41)	2164.92 (2025.00 to 2283.85)	−1.96 (−3.59 to −0.29)	48.28 (31.82 to 66.87)	47.24 (31.00 to 65.31)	−0.44 (−1.95 to 1.09)
Central Asia	6138.70 (5877.07 to 6337.12)	4796.57 (4365.40 to 5279.04)	−1.22 (−3.38 to 0.99)	68.16 (44.48 to 94.15)	67.92 (44.20 to 94.10)	−0.47 (−2.19 to 1.28)
Armenia	5602.53 (5311.61 to 5878.00)	3714.82 (3332.34 to 4157.57)	−2.84 (−4.97 to −0.65)	67.30 (44.14 to 92.18)	73.65 (48.16 to 100.75)	−0.57 (−2.33 to 1.22)
Azerbaijan	7088.72 (6633.53 to 7563.39)	5428.44 (4753.23 to 6126.12)	−1.20 (−4.07 to 1.76)	65.29 (42.96 to 90.96)	67.84 (44.20 to 93.31)	−0.28 (−1.90 to 1.38)
Georgia	6797.47 (6488.49 to 7083.63)	2504.60 (2243.74 to 2757.38)	−1.15 (−2.99 to 0.72)	73.62 (48.10 to 101.99)	60.64 (38.74 to 85.91)	−0.57 (−1.95 to 0.82)
Kazakhstan	5752.74 (5356.14 to 6172.92)	3897.28 (3497.79 to 4331.92)	−1.41 (−3.46 to 0.67)	74.29 (48.52 to 104.10)	73.22 (47.04 to 102.74)	−0.66 (−2.52 to 1.25)
Kyrgyzstan	5084.80 (4674.50 to 5496.05)	4720.32 (4026.70 to 5437.39)	−2.07 (−3.95 to −0.15)	65.05 (42.31 to 89.89)	60.16 (39.45 to 84.44)	−1.22 (−2.81 to 0.38)
Mongolia	5999.29 (5218.50 to 6876.59)	3825.01 (3286.54 to 4325.20)	−1.28 (−3.27 to 0.75)	68.13 (44.11 to 95.71)	67.29 (42.88 to 94.17)	−0.11 (−1.64 to 1.45)
Tajikistan	5898.09 (5241.48 to 6479.23)	4337.37 (3682.30 to 5048.74)	−1.38 (−3.52 to 0.81)	61.89 (39.33 to 87.61)	63.01 (41.14 to 89.15)	−0.40 (−1.94 to 1.17)
Turkmenistan	7796.74 (7453.50 to 8132.03)	6439.58 (5140.31 to 7948.40)	0.15 (−1.89 to 2.23)	67.09 (44.24 to 92.09)	73.12 (47.20 to 101.77)	0.48 (−1.23 to 2.22)
Uzbekistan	6059.89 (5754.17 to 6300.84)	6151.11 (5379.36 to 7002.04)	−0.77 (−3.41 to 1.94)	62.29 (40.20 to 86.17)	67.70 (44.65 to 93.83)	−0.13 (−1.97 to 1.75)

## Data Availability

The data that support the findings of this study are openly available in the Global Burden of Disease (GBD) database at http://vizhub.healthdata.org/gbd-results/.

## Abbreviations

GBD	Global Burden of Disease
IHD	Ischemic heart disease
ASPR	Age-standardized prevalence rates
ASIR	Age-standardized incidence rates
ASDR	Age-standardized death rates
DALY	Disability-adjusted life years
YLL	Years of life lost
YLD	Years lived with disability
AAPC	Average annual percent change
